# Conference Calendar

**DOI:** 10.1002/cfg.462

**Published:** 2005


					Comparative and Functional Genomics
Comp Funct Genom 2005; 6: 94-96.
Published online in Wiley InterScience (www.interscience.wiley.com). DOI: 10.1002/cfg.462
Conference Calendar
May -- July 2005
Animals
15th
International C. elegans Conference, June 25-29
http://genetics.faseb.org/genetics/Celegans/
4th
European Zebrafish Development and Genetics
Meeting, July 13-16
http://www.zebrafish-dresden.com/
Bioinformatics
9th
Annual Research in Computational Molecular Biology
(RECOMB 2005), May 14-18
http://www.broad.mit.edu/recomb2005/
BioIT World, May 17-19
http://www.bioitworldexpo.com/live/26/
IASTED -- Modelling and Simulation, May 18-20
http://www.iasted.org/conferences/2005/
cancun/ms.htm
CHI -- Cheminformatics, May 24-25
http://www.worldpharmacongress.com/
CHI -- Bioinformatics and Genome Research, June 13-14
http://www.beyondgenome.com/05bin.asp
World Congress in Applied Computing, June 20-23
http://www.world-academy-of-science.org/
WCAC2005/ws
ISCB -- 13th
Annual Intelligent Systems for Molecular
Biology (ISMB 2005), June 25-29
http://www.iscb.org/ismb2005/
International MultiConference in Computer Science &
Computer Engineering, June 27-30
http://www.world-academy-of-science.org/
IMCSE2005/ws
Functional Genomics
RNAi-2005: Chemical Biology to Bio-Drug & Therapeutic
Development, May 1-3
http://www.expressgenes.com/RNAi-2005-main.
htm
CHI -- RNAi for Pathway Analysis, May 16-18
http://www.healthtech.com/2005/rpa/index.asp
EuroSciCon: Identifying Gene Expression in Mammalian
Development and Disease, May 20
http://www.regonline.co.uk/eventinfo.asp?
EventId=16 304
CHI -- RNA Interference, June 13-14
http://www.beyondgenome.com/05rni.asp
Human Genomics and Genetics
ESHG -- European Human Genetics Conference, May
7-10
http://www.eshg.org/eshg2005/
Alan Wolffe EMBO Conference on Chromatin and
Epigenetics, May 19-22
http://www-db.embl.de/jss/EmblGroupsOrg/
conf 7
Mutation Detection 2005: VIII Intl. Symp. on Mutations in
the Genome, May 31-June 4
http://www.genomic.unimelb.edu.au/santorini.
html
GRC -- Human Genetics & Genomics, July 24-29
http://www.grc.uri.edu/programs/2005/
humangen.htm
General
IBC -- TIDES, May 1-5
http://www.ibclifesciences.com/tides
CHI -- Genomic and Proteomic Sample Preparation, May
4-5
http://www.healthtech.com/2005/smp/index.asp
Bio Nano Conference, May 8-12
http://www.nsti.org/BioNano2005/
CSHL -- The Biology of Genomes, May 11-15
http://meet1.cshl.edu/meetings/genome05.shtml
Copyright  2005 John Wiley & Sons, Ltd.
Conference Calendar 95
CHI -- Nucleic Acid-Based Technologies, June 27-28
http://www.healthtech.com/2005/pcr/index.asp
BS -- BioScience 2005, July 17-21
http://www.bioscience2005.org/
Metabolomics
GRC -- Plant Metabolic Engineering, July 10-15
http://www.grc.uri.edu/programs/2005/plant.
htm
GRC -- Enzymes, Coenzymes & Metabolic Pathways, July
17-22
http://www.grc.uri.edu/programs/2005/enzymes.
htm
Microbes
GRC -- Microbial Population Biology, July 17-22
http://www.grc.uri.edu/programs/2005/
micropop.htm
IUMS 2005: Microbes in a Changing World, July 23-28
http://www.iums2005.org/iums.asp
GRC -- Applied & Environmental Microbiology, July 24-29
http://www.grc.uri.edu/programs/2005/applied.
htm
Pharmacogenomics/Genomics and Disease
CHI -- Microarrays in Medicine, May 4-5
http://www.healthtech.com/2005/mar/index.asp
EuroMedLab 2005, May 8-12
http://www.glasgow2005.org/index.html
CLAS -- Molecular Basis of Health and Disease: Tools
Technologies and Applications, May 11-14
http://www.clasnewengland.org/national-
meeting.htm
CHI -- The World Pharmaceutical Congress, May 24-26
http://www.worldpharmacongress.com/
CHI -- The Biomarker World Congress, May 23-25
http://www.healthtech.com/2005/bmc/index.asp
IBC -- Drug Discovery & Development Asia Pacific, June
1-3
http://www.drugdisc.com/3110
GRC -- Toxicogenomics, June 5-10
http://www.grc.uri.edu/programs/2005/toxico.
htm
CHI -- Genomic Variation: Enabling Personalized
Medicine, June 15-16
http://www.beyondgenome.com/05gvr.asp
ESF-FG -- Personalized Medicine Europe: Health, Genes &
Society, June 19-21
http://www.functionalgenomics.org.uk/sections/
activitites/2005/Livshits/info.htm
Plants
5th
International Triticeae Symposium, June 6-10
http://www.vurv.cz/triticeae/
5th
Symposium on Post-Transcriptional Regulation of Plant
Gene Expression, June 8-12
http://research.cm.utexas.edu/kbrowning/
ptropge/
16th
International Conference on Arabidopsis Research,
June 14-19
http://www.union.wisc.edu/conferenceservices/
arabidopsis/
ASPB -- Plant Biology 2005, July 16-20
http://www.aspb.org/meetings/pb-2005/
Proteomics
6th
European Symposium of The Protein Society, April 30-
May 4
http://www.proteinsociety2005.info/barcelona.
html
CHI -- Difficult to Express Proteins, May 16-17
http://www.healthtech.com/2005/dpx/index.asp
CHI -- Phage Display for Engineering Protein
Therapeutics, May 16-17
http://www.healthtech.com/2005/pgd/index.asp
ASMS -- 53rd
Conference on Mass Spectrometry, June
5-9
http://www.asms.org/Default.aspx?tabid=43
Copyright  2005 John Wiley & Sons, Ltd. Comp Funct Genom 2005; 6: 94-96.
96 Conference Calendar
ESF-FG -- Ligand Binders Against the Proteome, June
13-15
http://www.functionalgenomics.org.uk/sections/
activitites/2005/Taussig/taussig.htm
CHI -- Proteomics, June 15-16
http://www.beyondgenome.com/05pro.asp
GRC -- Proteins, June 19-24
http://www.grc.uri.edu/programs/2005/proteins.
htm
30th
FEBS & 9th
IUBMB Congress: The Protein World, July
2-7
http://www.febs-iubmb-2005.com/
19th
Annual Symposium of The Protein Society, July
30-August 3
http://www.proteinsociety.org/pages/page00g.
htm
Structural Genomics
GRC -- Structural, Functional & Evolutionary Genomics,
June 19-24
http://www.grc.uri.edu/programs/2005/geno-
mics.htm
Systems Biology
CHI -- Systems Biology, June 15-16
http://www.beyondgenome.com/05isb.asp
Transcriptomics/Regulation
ESF-FG -- Transcription Networks: a Global View, May
26-28
http://cazorla.cnb.uam.es/esf/index.html
Biotech Industry
IBC -- Bio-Collaborations Asia, May 18-20
http://www.ibclifesciences.com/3109
BIO2005, June 19-22
http://www.bio.org/events/2005/
Key:
ASPB: American Society of Plant Biologists:
http://www.aspb.org/
ASMS: American Society for Mass Spectrometry:
http://www.asms.org/
BIO: Biotechnology Industry Organization:
http://www.bio.org/
BS: Biochemical Society:
http://www.biochemistry.org/
CHI: Cambridge Healthtech Institute:
http://www.healthtech.com
CLAS: Clinical Ligand Assay Society:
http://www.clasnewengland.org/
CSHL: Cold Spring Harbor Laboratories:
http://meetings.cshl.org/index.htm
ESF-FG: European Science Foundation Programme
in Functional Genomics:
http://www.functionalgenomics.org.uk/
ESHG: European Society of Human Genetics:
http://www.eshg.org/
EuroSciCon:http://www.euroscicon.com/
FEBS: Federation of European Biochemical Soci-
eties:
http://www.febs.unibe.ch/
GRC: Gordon Research Conferences:
http://www.grc.uri.edu/
IASTED: International Association of Science and
Technology for Development:
http://www.iasted.org/
IBC: IBC Conferences:
http://www.ibc-lifesci.com
ISCB: The International Society for Computational
Biology:
http://www.iscb.org/
IUBMB: International Union of Biochemistry and
Molecular Biology:
http://www.iubmb.unibe.ch/
IUMS: International Union of Microbiological
Societies:
http://www.iums.org/
The Conference Calendar is a listing of forthcoming conferences of interest to our readership. We provide
the title, dates and web address of each conference, to enable readers to find out more information.
Inclusion does not imply endorsement by the journal. If you know of a relevant conference, please
contact the Managing Editor.
Copyright  2005 John Wiley & Sons, Ltd. Comp Funct Genom 2005; 6: 94-96.

				

